# Performance of two different artificial intelligence models in dental implant planning among four different implant planning software: a comparative study

**DOI:** 10.1186/s12903-025-06336-0

**Published:** 2025-07-02

**Authors:** Pathompong Roongruangsilp, Walita Narkbuakaew, Pathawee Khongkhunthian

**Affiliations:** 1 Center of Excellence for Dental Implantology, Faculty of Dentistry, Chiang Mai University, Chiang Mai, Thailand; 2 National Electronics and Computer Technology Center, National Science and Technology Development Agency, Pathum Thani, Thailand

**Keywords:** Artificial intelligence, Machine learning, Deep learning, Dental implant, Cone-beam computed tomography

## Abstract

**Background:**

The integration of artificial intelligence (AI) in dental implant planning has emerged as a transformative approach to enhance diagnostic accuracy and efficiency. This study aimed to evaluate the performance of two object detection models, Faster R-CNN and YOLOv7 in analyzing cross-sectional and panoramic images derived from DICOM files processed by four distinct dental imaging software platforms.

**Methods:**

The dataset consisted of 332 implant position images derived from DICOM files of 184 CBCT scans. Three hundred images were processed using DentiPlan Pro 3.7 software (NECTEC, NSTDA, Thailand) for the development of Faster R-CNN and YOLOv7 models for dental implant planning. For model testing, 32 additional implant position images, which were not included in the training set, were processed using four different software programs: DentiPlan Pro 3.7, DentiPlan Pro Plus 5.0 (DTP; NECTEC, NSTDA, Thailand), Implastation (ProDigiDent USA, USA), and Romexis 6.0 (Planmeca, Finland). The performance of the models was evaluated using detection rate, accuracy, precision, recall, F1 score, and the Jaccard Index (JI).

**Results:**

Faster R-CNN achieved superior accuracy across imaging modalities, while YOLOv7 demonstrated higher detection rates, albeit with lower precision. The impact of image rendering algorithms on model performance underscores the need for standardized preprocessing pipelines. Although Faster R-CNN demonstrated relatively higher performance metrics, statistical analysis revealed no significant differences between the models (*p*-value > 0.05).

**Conclusions:**

This study emphasizes the potential of AI-driven solutions in dental implant planning and advocates the need for further research in this area. The absence of statistically significant differences between Faster R-CNN and YOLOv7 suggests that both models can be effectively utilized, depending on the specific requirements for accuracy or detection. Furthermore, the variations in imaging rendering algorithms across different software platforms significantly influenced the model outcomes. AI models for DICOM analysis should rely on standardized image rendering to ensure consistent performance.

## Background

Effective dental implant treatment planning significantly improves the success rate by facilitating the accurate three-dimensional positioning of the implant [1, 2]. Traditionally, dental implantologists frequently manage multiple Digital Imaging and Communications in Medicine (DICOM) file sets from Cone Beam Computed Tomography (CBCT) scan that require optimization. The process of three-dimensional treatment planning is often time-consuming and labor-intensive, particularly when performed sequentially by a single practitioner. Moreover, this workflow is largely dependent on the clinician’s expertise and subjective interpretation of the imaging data, which may introduce variability in diagnostic accuracy and treatment outcomes. The early image innovation about batch processing concepts enables the simultaneous and consistent manipulation of multiple medical images in a unified operation [3, 4].

Artificial intelligence (AI) technologies, particularly deep learning models, have emerged as promising tools. Recent systematic reviews highlight the growing potential of AI in various aspects of implant dentistry, including implant system identification, treatment planning, and outcome prediction. AI models have shown promising accuracy in detecting and classifying implant systems using panoramic and periapical radiographs [5], as well as in recognizing implant types, predicting success rates, and optimizing implant design [6]. Furthermore, AI applications have extended to automated detection of anatomical structures such as bone, maxillary sinuses, neural pathways, and teeth, supporting more precise implant planning [7]. Despite these advancements, the clinical integration of AI remains limited due to challenges such as the need for high-quality training data, lack of standardized protocols. Consequently, recent consensus about AI in dental research, has been proposed to highlight the need for well-designed studies with precise objective, reproducibility, and validation [8].

Deep learning-based object detection models, such as Faster Region Convolutional Neural Network (Faster R-CNN) and You Only Look Once (YOLO), have demonstrated promising potential. Their comparisons were demonstrated in Tables 1 and 2. Both models have shown promise in fields of dentistry [9–12]. Faster R-CNN, introduced by Ren et al. in 2015 [13], is an evolution of the earlier R-CNN and Fast R-CNN models. It integrates a Region Proposal Network (RPN) with a convolutional neural network, allowing it to generate and classify object proposals in a unified, end-to-end architecture. This two-stage detector innovation significantly improved detection speed and accuracy compared to its predecessors. Faster R-CNN has since become a benchmark model in medical imaging applications, particularly where high localization accuracy is needed [14, 15]. In addition, YOLOv7, developed by Wang et al. in 2022 [16], is a version 7 of the YOLO family of models, originally proposed by Redmon et al. in 2016 [17]. YOLOv7 introduces architectural innovations such as Extended Efficient Layer Aggregation Networks (E-ELAN) compound model scaling, and re-parameterization (REP) layer, these innovations enhance both detection precision and real-time performance. Unlike two-stage detectors, YOLOv7 performs detection in a single pass, making it well-suited for clinical environments where computational efficiency and speed are crucial. While YOLO models have been widely adopted in general computer vision tasks, their application in dental implantology using CBCT data remains underexplored.

**Table 1:** Comparative summary between Faster R-CNN and YOLOv7

Component	Faster R-CNN	YOLOv7
Detection Style	Two-stage	One-stage
Input	Image (resized while maintaining aspect ratio)	Image (resized, typically square)
Backbone	Typically, ResNet-50 or ResNet-101	Custom convolutional network using CBS, ELAN, and E-ELAN blocks
Feature Aggregation	No explicit neck; proposals generated directly from backbone features	PAN or FPN style neck for multi-scale feature fusion
Proposal Mechanism	Region Proposal Network (RPN) generates candidate object boxes	None – object predictions are made directly in a single pass
Region of Interest (RoI) Processing	RoI Align extracts fixed-size feature maps from proposals	None – all spatial locations are processed uniformly
Detection Head	Fully connected layers for classification and bounding box regression	Dense convolutional heads predict class, objectness, and box offsets for each anchor
Output	Final bounding boxes with class labels and confidence scores	Final bounding boxes with class labels and confidence scores
Speed	Slower – higher accuracy, but computationally intensive	Very fast – optimized for real-time performance
Model Size	Larger, due to separate RPN and fully connected layers	Lightweight to mid-size, depending on the version
Training Strategy	Standard loss functions for classification, regression, and RPN	Optimized with label assignment strategies, auxiliary heads, and efficient gradient flows

**Table 2:** A stage-by-stage architecture of Faster R-CNN and YOLOv7

Stage	Layer Type	Faster R-CNN	YOLOv7
1	Input	Image → Resize (maintaining aspect ratio)	Image → Resize (typically to square)
2	Backbone	ResNet (Conv + BN + ReLU + Pooling + residual)	ELAN/CBS blocks
3	Feature Aggregation	—	PANet-style neck
4	Proposal/Detection	RPN → Anchor Boxes	Detection heads at 3 scales
5	Region Processing	RoI Align → FC	Not used
6	Output	Class + BBox (via FC layers)	Class + Objectness + BBox (via conv heads)

By combining these object detection models with CBCT scan processing, repetitive tasks can be streamlined, providing automated and consistent evaluations. This integration reduces the workload for clinicians while improving accuracy and overall treatment outcomes. However, their performance still varies due to underlying methodology, and imaging modalities. Particularly, when dealing with DICOM images from various imaging software. Each software employs unique rendering algorithms that dictate the output images; resolution, contrast, and feature enhancement. These proprietary algorithms are often undocumented [18], resulting in inconsistencies in image representation. Even though the final image may appear identical to the human eye, it may affect the AI performance. These proprietary algorithms, often undocumented, resulted in inconsistencies in image representation. For instance, variations in anatomical features rendered across platforms may influence model detection and classification.

Although several AI-based tools have been proposed for dental imaging, The two main research questions have arisen. First, do different software result in the accuracy and detection rate due to the different algorithm of the Artificial Neural Network (ANN) of each AI? Second, can AI tool using a single software platform performing equally well across all CBCT systems? Moreover, to our best knowledge, there is no published study has specifically compared the performance of different deep learning models, such as Faster R-CNN and YOLOv7 in preoperative implant planning using CBCT data.

This study aims to evaluate and compare the performance of these models in analyzing cross-sectional and panoramic images generated by various DICOM imaging software. Furthermore, this study also investigates the impact of variability in rendering algorithms and proposes strategies for optimizing these models.

## Methods

### Study design and ethical approval

This comparative study was conducted in accordance with the Strengthening the Reporting of Observational Studies in Epidemiology (STROBE) guidelines for reporting observational research. Ethical approval was obtained from the Human Experimentation Committee, Faculty of Dentistry, Chiang Mai University, Thailand (Certification No. 30/2019).

### Data collection and inclusion/exclusion criteria

A total of 332 implant position images used in this study were derived from DICOM files obtained from 184 CBCT scans of patients treated at the Center of Excellence for Dental Implantology, Faculty of Dentistry, Chiang Mai University. These scans were collected over the period from 2013 to 2019, the selection criteria for CBCT scan were as followed:

Inclusion Criteria.

- CBCT scans of patients with missing posterior maxillary teeth (from the first premolar to the second molar) who require either single or multiple implant placements are included.
- Absence of image artifacts
- Well-formed dental arches
- Sufficient image quality for accurate analysis

Exclusion Criteria

- CBCT scans with poor image quality or artifacts
- Incomplete or distorted dental arches
- CBCT images lacking clear anatomical references

### Image processing and preparation

Three hundred DICOM reconstruction were generated using DentiPlan Pro 3.7 software (NECTEC, NSTDA, Thailand) to create input images, which consisted of two types of images:

1. Panoramic images are generated by defining an imaginary panoramic line in the axial view, which extends from the left condyle, passes through the center of each tooth, and terminates at the right condyle. This technique ensures a comprehensive view of the dental arch for detailed analysis.
2. Cross-sectional images are then generated at specific implant positions from panoramic image, ensuring proper implant position. For patients requiring multiple implants, individual cross-sectional images are created for each implant site for treatment planning.

**Fig. 1:**
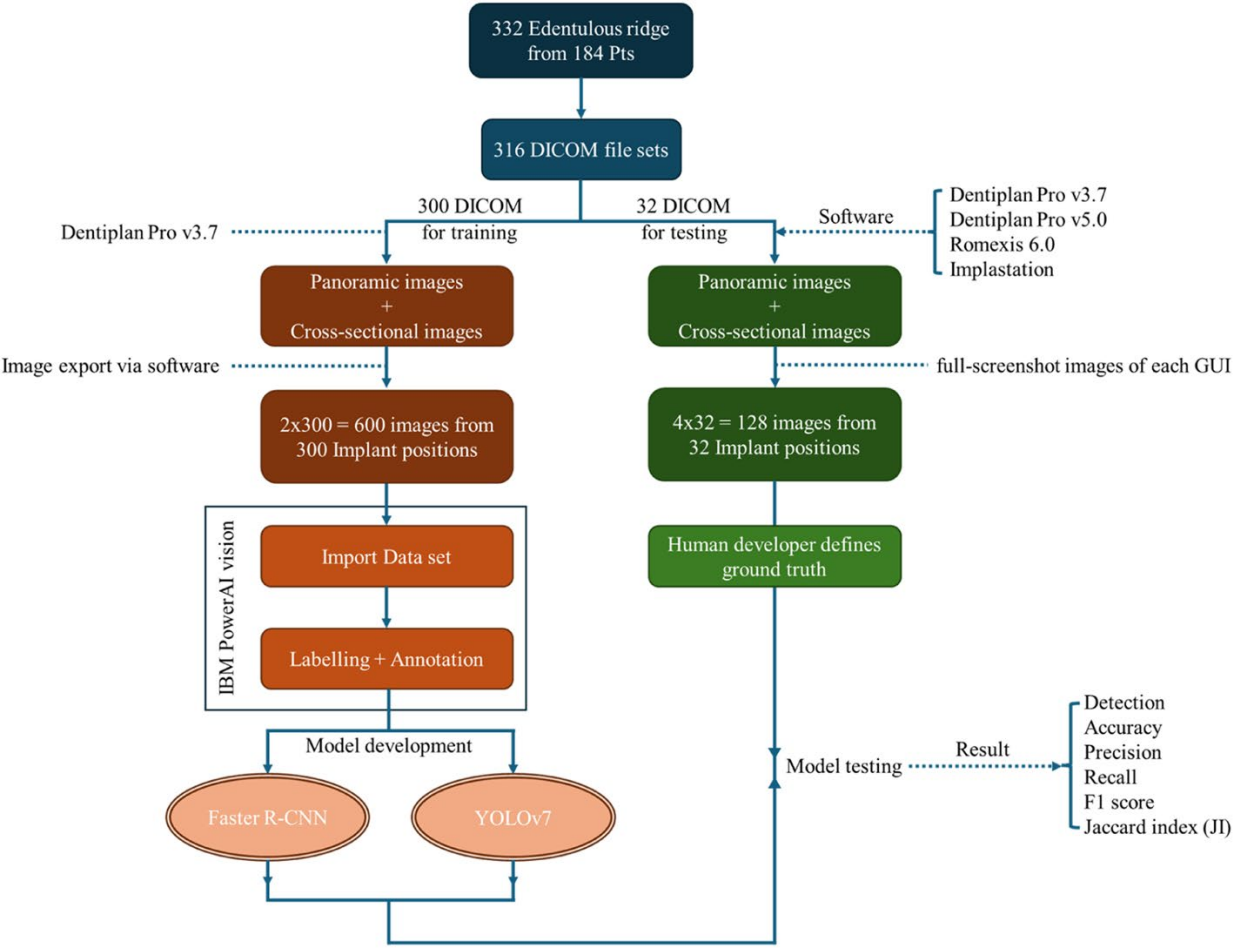
Conceptual framework of the study

**Fig. 2:**
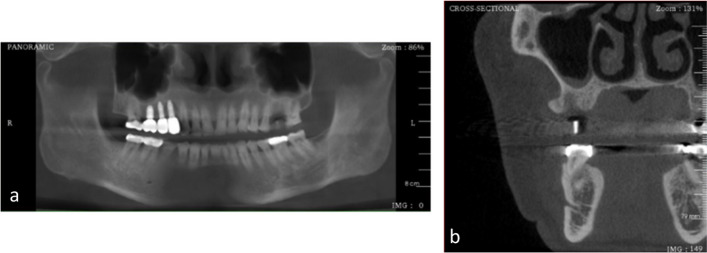
Export panoramic image (**a**) and cross-sectional image (**b**) of DentiPlan Pro version 3.7

The image sets of 300 implant positions were exported anonymously in Joint Photographic Experts Group (JPEG) format with 24-bit color depth (Figs. 1 and 2) which is a scene capture in RGB format from the manufacturer of the CBCT device. Therefore, all the displayed 8-bit grayscale images are recorded as a color image (24 bit) comprising red, green, and blue channels with identical values.

### Image annotation and labeling

The datasets were then organized for developing the object detection models. The labeling and annotation processes were performed on the IBM PowerAI Vision platform (IBM Thailand Co., Ltd., Thailand) by a single implantologist with extensive clinical experience to ensure consistency in labeling. The observer was evaluated using intra-calibration coefficient (ICC = 1).

**Fig. 3:**
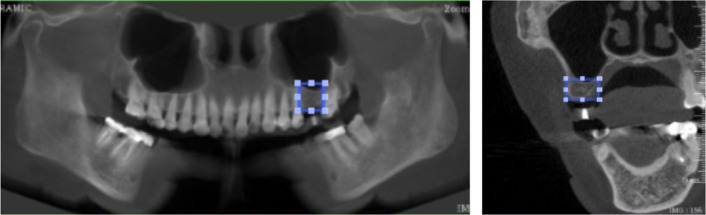
Example image labeling in panoramic image and cross-sectional image

In each image, the labeled region was delineated as a square by connecting four lines drawn from the outermost edges of the alveolar bone suitable for implant placement. In panoramic images, these four lines corresponded to the upper, lower, mesial, and distal borders, while in cross-sectional images, they corresponded to the upper, lower, buccal, and lingual borders. A visual representation of the labeling process is provided in Fig. 3.

**Fig. 4:**
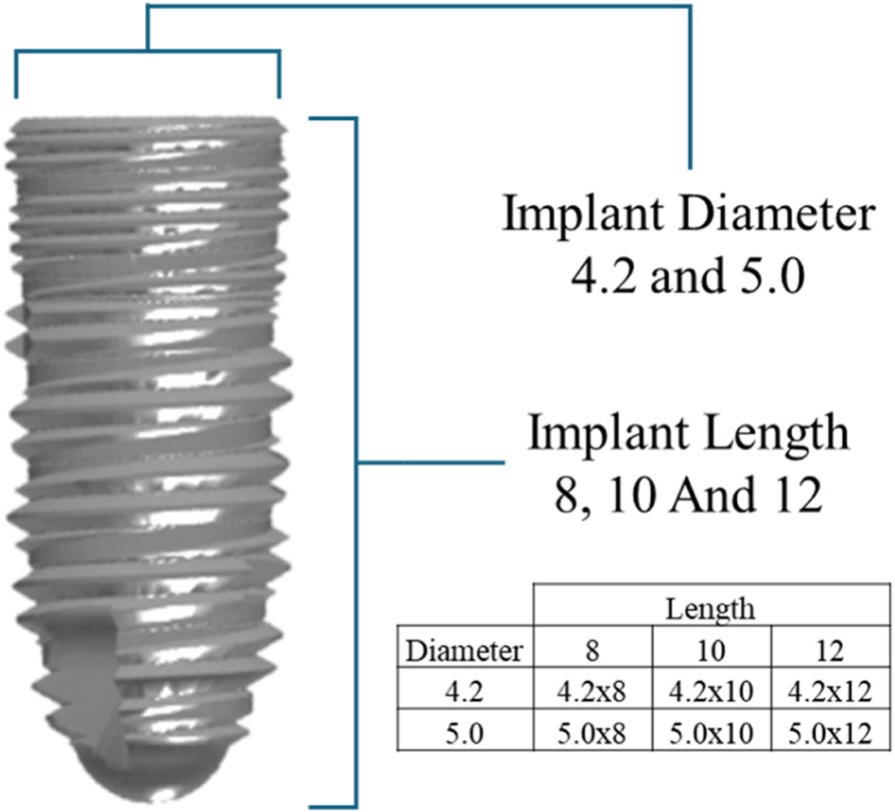
The available size of NOVEM dental implant system

The dental implant system used in this conditional programming was the NOVEM dental implant system (NOVEM®, Novem Innovations Co., Ltd., Chiang Mai, Thailand), the available diameter and length, which will be used in the posterior maxilla are demonstrated in Fig. 4

The implant selection was based on the available space, considering both bone width and bone height. Each labeled region was annotated with the appropriate implant size and technique, such as"Int SFE"for internal sinus floor elevation and"Lat SFE"for lateral sinus floor elevation. The annotation process adhered to the criteria established by Roongruangsilp and Khongkhunthian in 2021 [19].

Each labeled area was annotated with eight distinct categories: 4.2 × 8, 4.2 × 10, 4.2 × 12, 5.0 × 8, 5.0 × 10, 5.0 × 12, Int SFE (internal sinus floor elevation), and Lat SFE (lateral sinus floor elevation) for both panoramic and cross-sectional images. The annotations for panoramic and cross-sectional images were performed independently, with each labeled area marked using a corresponding bounding box.

### Development of AI models

Two object detection models were developed including faster R-CNN model and YOLOv7 model. Firstly, faster R-CNN model was developed in the IBM PowerAI Vision platform. Secondly, YOLOv7 was developed using the code from the official YOLO Github account [16]. The hyperparameters of both models were primarily configured as recommended by the AI developer and no further image adjustment had been performed. After both models were completely developed, they were subsequently deployed utilizing the Application Programming Interface (API) to facilitate the specific testing application. The model structure of the AI are showed in Tables 1 and 2.

### Model evaluation and testing

**Fig. 5:**
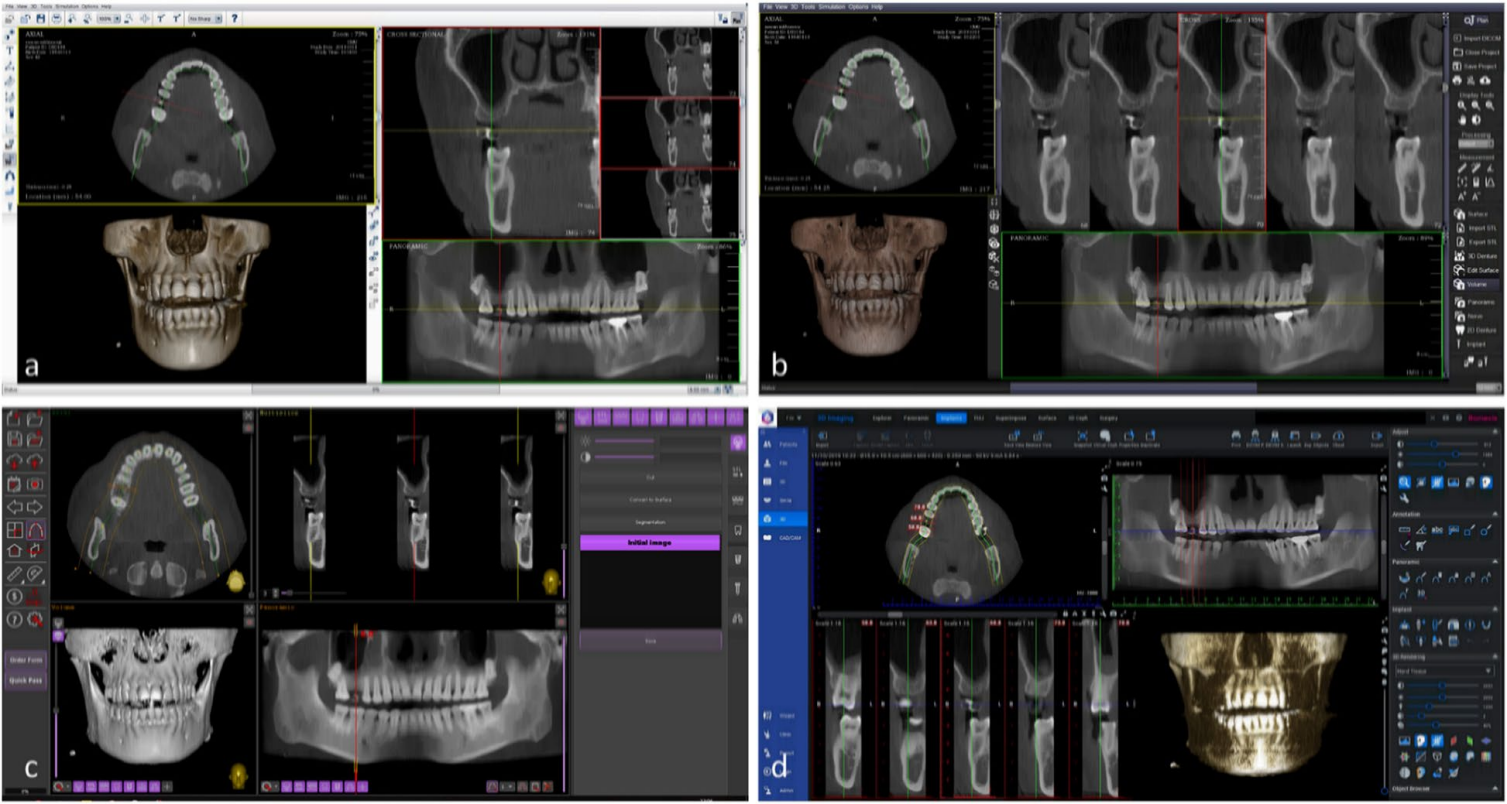
The full-screenshot images of GUI including DentiPlan Pro 3.7 (**a**), DentiPlan Pro Plus 5.0 (**b**), Implastation (**c**), and Romexis 6 (**d**)

The remaining 32 implant position images were designated as the testing set to evaluate the performance of the developed model. Importantly, none of the testing images were included in the model development phase, ensuring an unbiased evaluation. The DICOM files for these implant positions were reconstructed using four different software platforms: DentiPlan Pro 3.7, DentiPlan Pro Plus 5.0 (DTP; NECTEC, NSTDA, Thailand), Implastation (ProDigiDent USA, USA), and Romexis 6.0 (Planmeca, Finland). Two software are belonged to the CBCT device with different version (DentiPlan Pro 3.7 and DentiPlan Pro Plus 5.0), one is a commercialized software (Romexis 6.0), and another one is free software from internet (Implastation). These software were utilized to generate the aforementioned two types of planning images. Full-screenshot images of each software interface were anonymously captured to ensure the protection of private information. The captured images were stored in the JPEG format at a resolution of 1920 × 1080 pixels with 24-bit depth (Fig. 5).

**Fig. 6:**
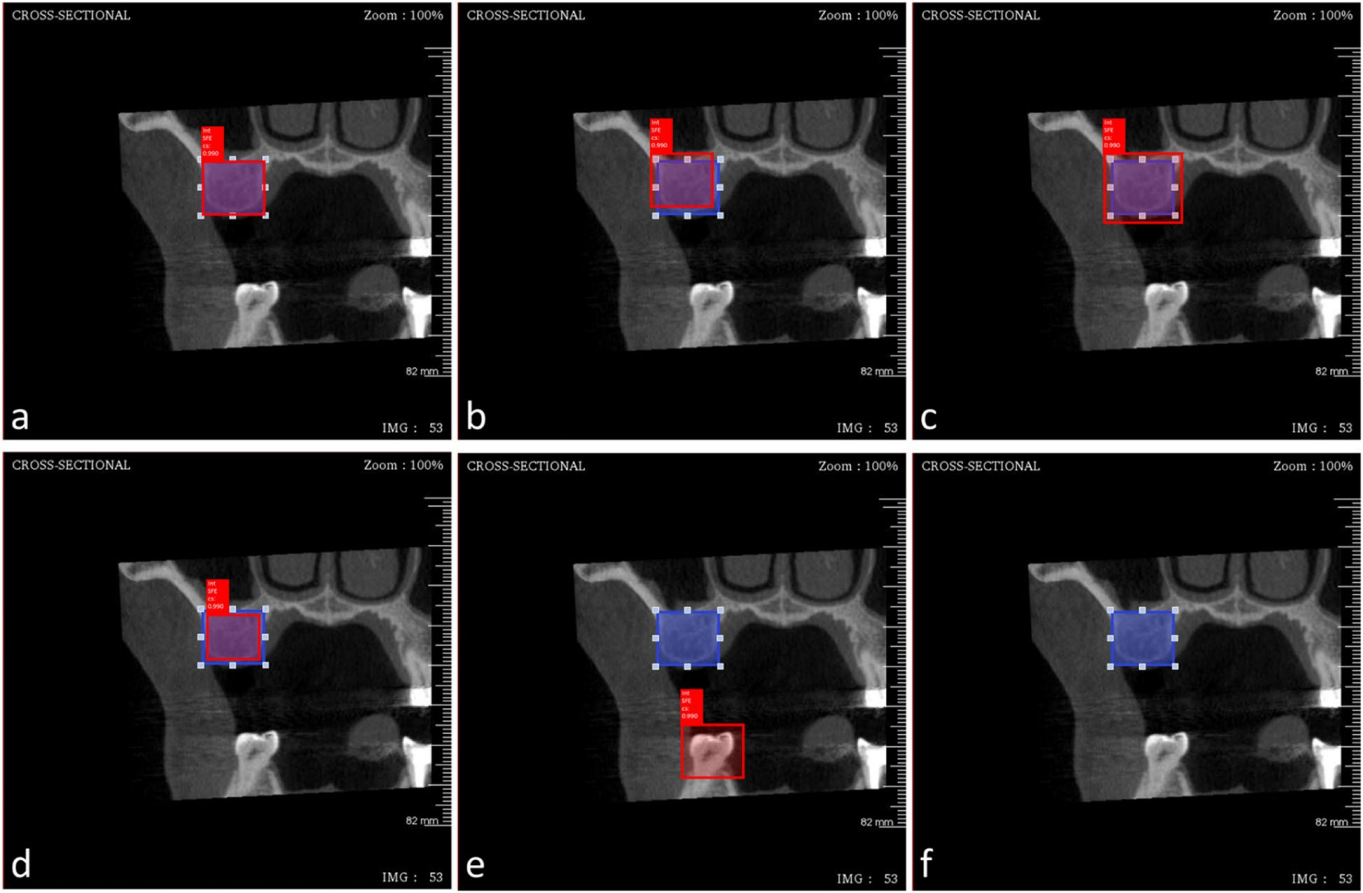
The six examples of all potential outcome displayed the comparison between predicted bounding box (red square) and ground truth bounding box (blue square) including complete matching (**a**), intersection matching (**b**, **c**, and **d**), and no detection (**e**, and **f**)

The same implantologist who labeled and annotated the training images also performed the same procedure on each testing image to establish the ground truth, which served as the benchmark for evaluating the model’s performance. During the testing process, images were uploaded individually, and the deployed model displayed its detections with bounding boxes, annotations, and confidence percentages as demonstrated in Fig. 6.

Object detections with intersection over union (IoU) over 0.75 with a confidence annotation threshold exceeding 0.95 were recorded for further comparison with ground truth. If the IoU was less than 0.75 or a confidence annotation threshold was less than 0.95, it would be classified as no detection.

The threshold selection criteria were adapted from the AP@0.75 metric which is commonly used in object detection benchmarks [20, 21], focusing on precision at stricter spatial overlap (IoU > 0.75). This higher threshold was chosen to better reflect the stringent accuracy required for anatomical localization in dental implant planning, where even minor deviations can influence treatment outcomes. The confidence threshold of 0.95 was applied to prioritize high-certainty predicted annotation, aligning with clinical standards that generally tolerate a maximum of 5% error. This conservative thresholding approach supports the reliability and reproducibility of model outputs in a real clinical context.

All detections that covered the predefined criteria were collected, and comparative analysis of the corresponding annotations were then assessed using various metrics, including detection rate, accuracy, precision, recall, F1 score, and Jaccard index (JI), depending on the dataset's characteristics and suitability for each metric. These metrics were analyzed in both the panoramic and cross-sectional images, and the final calculated data were then incorporated into the model’s outcome. For better understanding, the equation of each metric was demonstrated below.

$$Detection\;rate=\frac{number\;of\;detection\;events\;during\;the\;test\;process}{number\;of\;the\;total\;images\;used\;in\;the\;test\;process}$$

$$Accuracy=\frac{TP+TN}{All\;detection\;events}$$

$$Precision= \frac{TP}{TP+FP}$$

$$Recall= \frac{TP}{TP+FN}$$

$$F1\;score=2\times\frac{Precision\times Recall}{Precision+Recall}$$

$$Jaccard\;Index\left(JI\right)=\frac{TP}{TP+FP+FN}$$

TP and TN were defined as true positive and true negative, respectively (correct predictions for category). FP was defined as false positive (incorrect predictions assigned to category). FN was defined as false negative (missed predictions for category).

### Statistical analysis

The Shapiro–Wilk test was utilized to assess the normality of the differences in metrics between Faster R-CNN and YOLOv7 across cross-sectional and panoramic images. Subsequently, the Wilcoxon signed-rank test and paired t-tests were conducted to compare the differences in these metrics across the two images types and detection models.

## Results

**Fig. 7:**
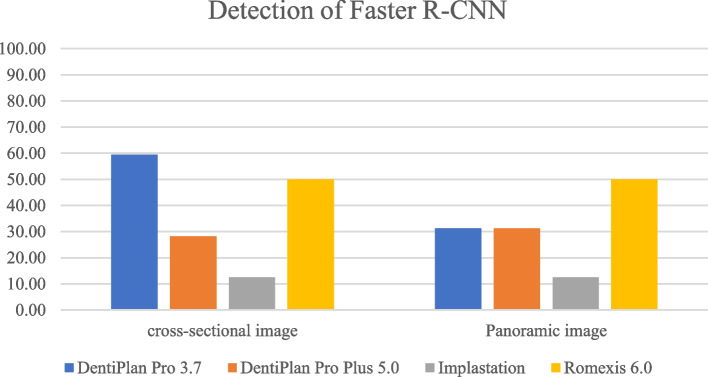
The detection of Faster R-CNN

**Fig. 8:**
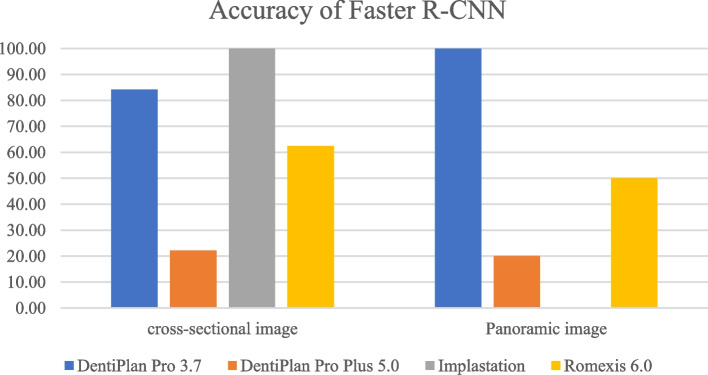
The accuracy of Faster R-CNN

**Fig. 9:**
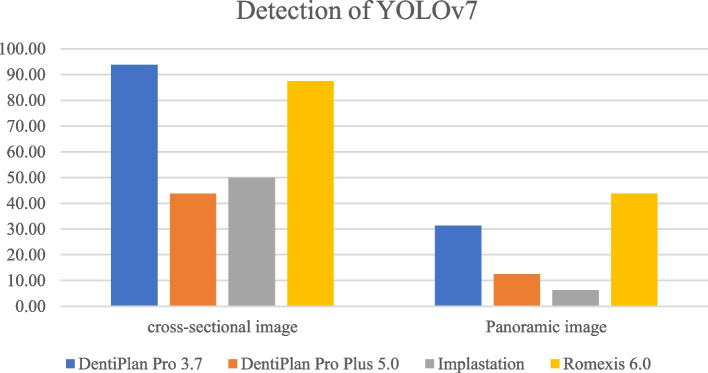
The detection of YOLOv7

**Fig. 10:**
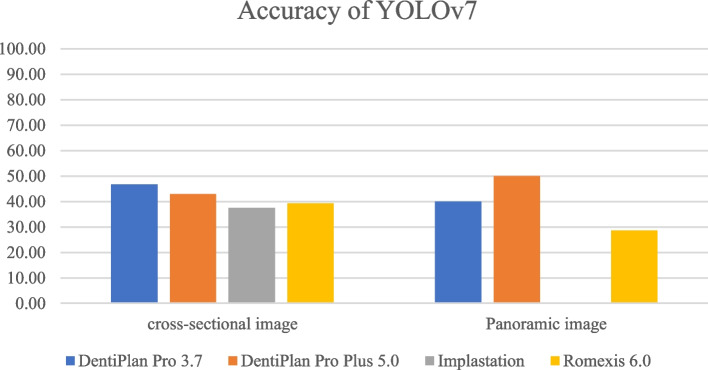
The accuracy of YOLOv7

The distribution of 300 DICOM file sets used in the training process was displayed in Table 3. The detection and accuracy of faster R-CNN and YOLOv7, as tested by four different imaging software were displayed in Tables 4 and 5 and Figs. 7, 8, 9 and 10. The metrics such as precision, recall, F1 score, and Jaccard index, were demonstrated in Tables 6 and 7.

**Table 3:** The distribution of each DICOM file sets used in the training process

Classification	No. of cases
4.2x8	13
4.2x10	17
4.2x12	61
5.0 × 8	23
5.0 × 10	20
5.0 × 12	47
Int SFE	55
Lat SFE	64

**Table 4:** The detection and accuracy of Faster R-CNN in cross-sectional and panoramic images among four different software

		Testing images obtained from the following software			
		DentiPlan Pro 3.7	DentiPlan Pro 5.0	Implastation	Romexis 6.0
Cross-sectional image	detection	59.38	28.13	12.50	50.00
	accuracy	84.21	22.22	100.00	62.50
Panoramic image	detection	31.25	31.25	12.50	50.00
	accuracy	100.00	20.00	0.00	50.00

**Table 5:** The detection and accuracy of YOLOv7 in cross-sectional and panoramic images among four different software

		Testing images obtained from the following software			
		DentiPlan Pro 3.7	DentiPlan Pro 5.0	Implastation	Romexis 6.0
Cross-sectional image	detection	93.75	43.75	50.00	87.50
	accuracy	46.67	42.86	37.50	39.29
Panoramic image	detection	31.25	12.50	6.25	43.75
	accuracy	40.00	50.00	0.00	28.57

**Table 6:** Precision, recall, F1 score, and Jaccard index (JI) of Faster R-CNN

Class	Cross-Sectional Image				Panoramic Image			
	Precision	Recall	F1	JI	Precision	Recall	F1	JI
4.2 × 8	0.00	0.00	N/A	0.00	0.00	N/A	N/A	0.00
4.2 × 10	0.00	N/A	N/A	0.00	0.00	0.00	N/A	0.00
4.2 × 12	100.00	61.54	76.19	61.54	100.00	25.00	40.00	25.00
5.0 × 8	0.00	N/A	N/A	0.00	50.00	100.00	66.67	50.00
5.0 × 10	80.00	80.00	80.00	66.67	100.00	66.67	80.00	66.67
5.0 × 12	100.00	100.00	100.00	100.00	100.00	100.00	100.00	100.00
Int SFE	80.00	50.00	61.54	44.44	0.00	0.00	N/A	0.00
Lat SFE	100.00	90.91	95.24	90.91	50.00	60.00	54.55	37.50

^*^N/A referred to “Not Available” as the metrics cannot be calculated due to a lacked of detected cases by the model

**Table 7:** Precision, recall, F1 score, and Jaccard index (JI) of YOLOv7

Class	Cross-Sectional Image				Panoramic Image			
	Precision	Recall	F1	JI	Precision	Recall	F1	JI
4.2 × 8	0.00	N/A	N/A	0.00	0.00	N/A	N/A	0.00
4.2 × 10	0.00	100.00	0.00	0.00	0.00	N/A	N/A	0.00
4.2 × 12	100.00	33.33	50.00	33.33	N/A	0.00	N/A	0.00
5.0 × 8	0.00	0.00	N/A	0.00	66.67	100.00	80.00	66.67
5.0 × 10	0.00	0.00	N/A	0.00	N/A	N/A	N/A	N/A
5.0 × 12	75.00	75.00	75.00	60.00	N/A	0.00	N/A	0.00
Int SFE	75.00	60.00	66.67	50.00	100.00	25.00	40.00	25.00
Lat SFE	62.50	100.00	76.92	62.50	40.00	100.00	57.14	40.00

^*^N/A referred to “Not Available” as the metrics cannot be calculated due to to a lacked of detected cases by the model

The Shapiro–Wilk test was used for the normality test of the differences of metrics in cross-sectional and panoramic images between Faster R-CNN and YOLO. In the cross-sectional image, recall and JI differences appeared normally distributed (*p*-value = 0.2695 and 0.1396, respectively) while precision and F1 score differences did not appear to follow a normal distribution (*p*-value = 0.0055 and 0.0164, respectively). In the panoramic image, the results suggested that the differences in metrics like precision, recall, F1 score, and JI between Faster R-CNN and YOLOv7 follow a normal distribution with *p*-values of 0.1423, 0.3681, 0.3003, and 0.1211, respectively.

Overall, Faster R-CNN outperformed YOLOv7 across all metrics, as shown in Tables 8 and 9 for each macro-averaged metric. In the cross-sectional analysis, the precision and F1 score differences did not exhibit a normal distribution. Therefore, the Wilcoxon signed-rank test was considered more appropriate for these metrics. In contrast, the differences for the remaining metrics adhered to a normal distribution, permitting the use of paired t-tests for comparison between Faster R-CNN and YOLOv7.

**Table 8:** Mean, variance and *p*-value of individual metrics; precision, recall, F1 score, and Jaccard index (JI) in cross-sectional image between faster R-CNN and YOLOv7

	Cross-Sectional Image							
	Precision		Recall		F1		JI	
	Faster R-CNN	YOLOv7	Faster R-CNN	YOLOv7	Faster R-CNN	YOLOv7	Faster R-CNN	YOLOv7
Mean	57.50	39.06	47.81	46.04	51.62	33.57	45.44	25.73
Variance	2335.71	1849.89	1809.74	1911.06	1963.51	1352.72	1707.05	831.63
*p*-value	0.0679^+^		0.9243^*^		0.1250 ^+^		0.0653^*^	

^+^Wilcoxon signed rank test was used to compare the metrics between Faster R-CNN and YOLOv7

^*^Paired t-test was used to compare the metrics between Faster R-CNN and YOLOv7

**Table 9:** Mean, variance and *p*-value of individual metrics; precision, recall, F1 score, and Jaccard index (JI) in panoramic image between faster R-CNN and YOLOv7

	Panoramic Image							
	Precision		Recall		F1		JI	
	Faster R-CNN	YOLOv7	Faster R-CNN	YOLOv7	Faster R-CNN	YOLOv7	Faster R-CNN	YOLOv7
Mean	50.00	25.83	43.96	28.13	42.65	22.14	34.90	16.46
Variance	2142.86	1529.37	1887.25	2042.41	1552.35	1048.98	1319.13	643.20
*p*-value	0.3704^*^		0.3698^*^		0.2700^*^		0.2698^*^	

^*^Paired t-test was used to compare the metrics between Faster R-CNN and YOLOv7

## Discussion

This study developed faster R-CNN and YOLOv7 models for dental implant treatment planning using multiple imaging software, such as DentiPlan, Implastation, and Romexis. These models would detect features within cross-sectional and panoramic images from DICOM file sets across varied imaging rendering algorithms. The results provided a comparative analysis of the performance of both models utilizing various metrics, including detection, accuracy, precision, recall, F1 score, and Jaccard index.

Although both model developments incorporated images from Dentiplan Pro 3.7 to ensure a consistent basis, their distinct methodological analyses resulted in varying performance outcomes. Faster R-CNN demonstrated a different response compared to YOLOv7 in cross-sectional image, exhibiting lower detection rate (59.38%) but higher accuracy (84.21%), whereas YOLOv7 achieved higher detection rate (93.75%) but lower accuracy (46.67%). This difference reflects the trade-offs inherent in the design of each model. YOLOv7 was more aggressive in detecting objects, but many of these detections were likely false. YOLO’s single-stage detection design focuses on speed and real-time detection often comes at the cost of precision. Faster R-CNN prioritizes accuracy due to its two-stage detection process. The RPN allows it to focus on detailed regions for higher accuracy [13, 17, 22], making it more suitable for applications where false positives can have significant consequences.

However, Faster R-CNN and YOLOv7 demonstrated identical detection rates (31.25%) in panoramic images, their accuracy differed significantly, with Faster R-CNN achieving 100.00% accuracy compared to 40.00% for YOLOv7. The result suggested that the panoramic images posed a similar level of difficulty in detection for both models. This might be due to challenges like larger field of view, overlapping anatomical structures, or lower resolution. The contrast in accuracy highlights Faster R-CNN’s robustness in challenging imaging conditions, where precision is vital, even if fewer detections are made. YOLOv7’s performance in panoramic imaging reveals limitations in handling complex or large-scale visual data effectively.

Each DICOM viewer software employed a distinct image rendering algorithm, these algorithms determine critical factors such as resolution, contrast, noise reduction, and feature enhancement. Differences in rendering can profoundly influence how detectable specific structures appear in the final images, which had a significant impact on the model performances.

In our study, Faster R-CNN demonstrated variable detection performance across different DICOM viewers software. Its performance also varied across imaging modalities, with notably higher detection in cross-sectional images compared to panoramic views. The highest detection was observed in DentiPlan Pro 3.7 (59.38%) and Romexis 6.0 (50.00%) for cross-sectional images, while performance dropped substantially in Implastation (12.50%). Interestingly, despite the low detection in Implastation, the model returned 100% accuracy. This suggests that the changes in image pre-process might have reduced the visibility of key anatomical features, leading to fewer detections. Nonetheless, implastation indicates that its rendering algorithm enhances the precision of feature representation for both models, reflecting a trade-off where fewer detections are achieved but with higher accuracy. Faster R-CNN suggests a conservative prediction approach, where the model may only respond to highly confident features but leads to a high rate of missed cases. Conversely, DentiPlan Pro 5.0 exhibited both low detection (28.13%) and poor accuracy (22.22%), which may be attributed to alterations in image rendering in the updated software version that possibly hindered the region proposal mechanism of Faster R-CNN.

However, YOLOv7 generally exhibited superior detection rates in cross-sectional images, particularly with DentiPlan Pro 3.7 (93.75%) and Romexis (87.50%) and this came at the expense of lower accuracy, ranging between 37.50% and 46.67%. the result indicated a higher false-positive rate. The YOLOv7 model’s architecture, which prioritizes sensitivity, likely contributed to this higher incidence of false positives. Moreover, DentiPlan Pro 5.0 and Implastation showed weaker detection rates (43.75% and 50.00%, respectively), but their accuracy still hovered below 45%, confirming that the model struggled to localize available implant positions correctly in these DICOM viewers software.

In panoramic images, detection rates for both Faster R-CNN and YOLOv7 declined across most software platforms. Notably, only Romexis 6.0 enhanced detection performance, yielding the highest rates with Faster R-CNN (50.00%) and YOLOv7 (43.75%). In contrast, the other software demonstrated considerably lower detection rates, ranging from 6.25% to 31.25%. Although DentiPlan Pro 3.7 showed a relatively low detection rate (31.25%), it achieved 100% accuracy with Faster R-CNN. This suggests that Faster R-CNN may function as a high-precision model for identifying available implant positions in panoramic images, though it appears to benefit less from alternative image rendering algorithms compared to other platforms.

These findings highlight the trade-off between sensitivity and precision across models and platforms. Faster R-CNN offers conservative, high-precision detection at the cost of sensitivity, while YOLOv7 exhibits high recall but reduced precision due to an increased number of false positives. This illustrates the inherent balance between sensitivity and precision, where one model prioritizes accuracy in its predictions, while the other emphasizes detection coverage at the expense of false positives. Furthermore, a model trained on images from one DICOM viewer software might struggle when presented with images processed by a different algorithm, reflecting a lack of generalization to variations in preprocessing. This suggests that AI models for DICOM analysis should consistently use a single image rendering algorithm throughout the development process. Variations in preprocessing, such as resolution, contrast, and noise reduction, can alter key image features, which may lead to inconsistent results or reduced model performance. Clinicians and developers must consider such factors when implementing AI tools in real-world settings. Single standardization of DICOM rendering and image protocols including uniform contrast settings, export formats, and image resolutions may be critical for maximizing the utility of AI in dental diagnostics and ensuring reliable, accurate predictions across different imaging platforms.

Other recent studies have explored the application of deep learning in dental implant planning, with a primary focus on quantitative measurements for dental planning rather than the available space for dental implant. Widiasri et al. (2022) [23]. implemented a YOLO-based model to identify the alveolar bone and mandibular canal in coronal CBCT slices, achieving a high precision rate of 99.46%. Their A two-way analysis of variance showed no statistically significant differences between AI and radiologist measurements with *p*-values of 0.249 for bone height and 0.184 for bone width, supporting the potential of YOLO models in diagnostic planning. Similarly, Bayrakdar et al. (2021) [24] evaluated a CNN-based system for implant site assessment in CBCT scans, The Wilcoxon signed-rank test revealed no statistically significant differences between AI and manual measurements for bone height in the premolar region of the mandible and the premolar and molar regions of the maxilla (*p*-values > 0.05). Conversely, statistically significant differences were observed between AI and manual measurements for bone width across all regions of the maxilla and mandible (*p* < 0.001). Furthermore, the paired differences analysis indicated no statistically significant differences in bone height measurements between the AI system and manual methods in the molar regions of the mandible and maxilla (*p*-value = 0.126 and *p*-value = 0.938, respectively) though significant differences were observed for bone thickness in every region (*p*-values < 0.001). While both studies underscore the reliability of deep learning models for morphometric evaluations, they focus primarily on measurement accuracy.

In contrast, this study extends previous research by evaluating AI performance using detection-based methods across different DICOM viewer software, this aspect was not addressed in earlier literature. Rather than measuring bone dimensions, this study targeted the spatial localization of dental implants, applying object detection models (Faster R-CNN and YOLOv7) across cross-sectional and panoramic images generated by various software platforms. This approach allows for a comprehensive assessment of how software variability influences model performance, an important consideration for clinical application that was not the primary focus in previous studies. Collectively, our work bridges the gap between detection accuracy and clinical applicability by emphasizing not only AI performance but also software interoperability. While earlier studies confirmed the potential of AI for measurement precision, our findings highlight the challenges of maintaining detection consistency across varied imaging environments. This distinction positions our study as a complementary and necessary step toward standardizing AI deployment in diverse clinical workflows.

Based on these findings, this study successfully developed an AI model focused on dental implant treatment planning in the posterior maxillary region, with related studies confirming its applicability solely to the posterior region. It can be inferred that the AI system is currently applicable only to this area. Developing an AI model tailored to the anterior region could provide significant benefits. Such a model could address current challenges by incorporating specific feature extraction algorithms and training on datasets enriched with anatomical variations from the anterior region. This targeted approach may enhance clinical utility for dental implant treatment planning in complex anatomical areas.

The performance of our developed models was relatively limited, indicating that further development is necessary before clinical application could be considered. This study has several limitations. Variability in imaging protocols and rendering algorithms across different DICOM viewer software may affect the generalizability of the AI models. Therefore, implementing a standardized preprocessing pipeline or developing adaptable models is essential to ensure consistent AI performance. Additionally, the limited dataset size and lack of diversity constrained the model’s ability to generalize across various patient demographics and anatomical variations. Another possible limitation is the use of only one expert for annotation during both training and testing phases. Future research should include larger datasets by investigating a broader range of clinical conditions and populations. Multi-center studies and validation using diverse datasets and multiple expert annotations may enhance the clinical applicability of AI-driven approaches in dental practice.

Our study focused on dental implant planning in the posterior maxilla using two AI models across 4 different DICOM viewers software. This model can assist clinicians during the initial evaluation phase of dental implant treatment planning; however, final decisions should still be confirmed independently by the clinician. When adopting such models in practice, it is advisable to prioritize alignment with specific clinical use-case requirements over marginal differences in model performance. The necessity of software standardization is also required.

## Conclusions

Within the limitation of this study, Faster R-CNN and YOLOv7 models were successfully developed for dental implant treatment planning using multiple DICOM imaging software. By utilizing cross-sectional and panoramic images, the models exhibited distinct performance. Although no statistically significant differences were observed between Faster R-CNN and YOLOv7, each model showed strengths in different areas: Faster R-CNN achieved higher accuracy, while YOLOv7 demonstrated superior detection capabilities. Notably, the variations in imaging rendering algorithms across software significantly impacted model outcome. Therefore, it may be concluded that the Al model for DICOM analysis should consistently employ with a single image rendering algorithm throughout the development process.

## Data Availability

The research data are available on resonable request to corresponding author.

## Abbreviations

DICOM: Digital Imaging and Communications in Medicine
CBCT: Cone Beam Computed Tomography
AI: Artificial intelligence
Faster R-CNN: Faster Region Convolutional Neural Network
YOLO: You Only Look Once
YOLOv7: YOLO version 7
E-ELAN: Extended Efficient Layer Aggregation Networks
REP: Re-parameterization
STROBE: Strengthening the Reporting of Observational Studies in Epidemiology
JPEG: Joint Photographic Experts Group
Int SFE: Internal sinus floor elevation
Lat SFE: Lateral sinus floor elevation
API: Application Programming Interface
IoU: Intersection over union
JI: Jaccard index
TP: True positive
FP: False positive
FN: False negative
